# Intelligence

**Published:** 1832-12

**Authors:** 


### INTELLIGENCE.

**MAGISTRATES *cannot compel* MEDICAL MEN *to certify without a Fee.***

A FEMALE was wounded by a stone thrown at another person: she was taken to the hospital. The mayor desired the surgeon in attendance to send him a certificate of his patient's condition; he declined to do so, unless he was paid for his trouble. His worship was angry, and wrote to the government on the subject: the following is the answer to his application.

"*Dublin Castle; Nov.* 13, 1832.

"Sir: I have received your letter of the 7th instant, with respect to a refusal, on the part of the hospital surgeon at Limerick, to give, without a fee, a certificate of the state of a girl, named Mary Noonan, who had been seriously injured by a stone thrown by Jeremiah Aherr at another person; and having, by the Lord Lieutenant's desire, referred your communication to the law officers of the Crown, I am to acquaint you, for the information of the magistrates at petty sessions, that he is not aware of any law which obliges the surgeon of an infirmary to give an opinion in cases of this nature.

"I have the honour to be, sir, &c.

"E. J. Stanley."

---

*Arrival in Paris of* M. Clot *and the* Egyptian Pupils; *interesting Communication to the Académie de Médecine.*

Our readers have probably already heard of the successful progress which medicine is making in Egypt. The Académie de Médecine in Paris was last week the scene of some highly interesting disclosures from M. Clot, relative to this subject. The session of the 13th of November was appointed by the Academy for the public reception of the mission which has lately arrived from that country, and we are enabled to present the profession with an original report of the proceedings. At the opening of this deeply-interesting meeting, the two most elevated and central benches of the academic precinct were occupied by twelve young Egyptians, who have been sent to France to pursue the study of medicine. In the midst of these young men, and distinguished by his brilliant costume, was M. Clot, the chief physician of the armies of Egypt, the founder and director of the school of Abouzabel, a Frenchman by birth, but promoted for his merit and labours to the ranks of Colonel and Bey. All eyes were turned in fixed attention on the extraordinary scene the Academy presented on this occasion.

The young men brought to Paris by M. Clot were selected from the school of Abouzabel, in which two of them are already professors. Their sombre figures and marked countenances formed a striking contrast with the surrounding assembly. The costume of the officers was red, richly embroidered with gold; and the assistants, sub-assistants, and major surgeon, bore marks of distinction of rank. A red Grecian bonnet formed their head dress. The pupils were habited in blue garments of simple fashion. M. Clot, who was also dressed in scarlet, wore a head-dress composed of a magnificent shawl turban of cachemire, a superb damasc girdle, and brilliant stars of diamonds on his breast. His complexion and figure had become so completely oriental, that those who even knew him before he left France could with difficulty recognise him. The Academy waited impatiently the recital of his adventurous labours, of the means by which he succeeded in transplanting European medical pursuits into a barbarous country, to the language, customs, and religion, of which he was a stranger. On the invitation of the president, M. Clot, after apologizing for the difficulty he experienced in addressing a French assembly, gave an account of his career in the following terms:

"I was an inhabitant of Marseilles, in which city I had practised medicine for several years, when an agent of the viceroy of Egypt made me proposals to proceed to that country to organize the service of health. Proceeding thither, in January 1825, with some companions, I was first of all employed in the organization of the milltary medical department. At that time the regular troops of the pacha amounted to about 25,000 men, and occupied Lower Egypt and the Morea. Their officers of health were persons of the lowest descriptions: shoemakers and bakers, who had consecutively become infirmary attendants, apothecaries, and physicians, without any examination or any proof of capacity or knowledge. The pacha testified his desire to have his service organized as it is in France; a wish which corresponded completely with my own.

"I first proposed the formation of a superior council of health, composed of three persons, namely, the chief physician of his highness, another physician of the court, and the pacha's private medical attendant. I was not myself a member. The government was satisfied with giving me the absurd title of physician in chief to the forces; a title which possessed no importance or reality except in time of war. An apothecary in chief to the forees was also created. This being done, I suggested that all the officers of health should be obliged to undergo an examination, and that those should be rejected who did not afford proofs of competency in the treatment of diseases. This measure, as may be imagined, procured me numerous enemies, and the speedy rejection of a number of ignorant candidates created such animosity against me, that my life was attempted by an assassin in the amphitheatre. Regimental hospitals were also created. I obtained for the medical officers the full use of military insignia, and the enjoyment of military rank and prerogatives. The army of the pacha being soon after augmented to 60,000 men, officers of health were wanting in proportion. To meet this want, I proposed to transform into a school of instruction the hospital of Abouzabel, situated near Heliopolis, and built on the ruins of an old barrack. I summoned to this seminary the best-informed of the rising generation, and one hundred young Arabs were the first pupils. New difficulties now presented themselves. We were mutually ignorant of each other's language: how then, I said, were the pupils to be instructed? I fortunately succeeded in finding in Paris three persons who understood French, Italian, and Arabic; but these persons were destitute of medical knowledge. I told them they should become physicians, but that they should first be pupils. I accordingly set them to work on the translation of a treatise on anatomy, lessons from which were dictated to the pupils, who were afterwards examined by means of the interpreters. But plates and wax figures having failed to communicate the necessary anatomical knowledge, the dissection of the actual subject became essential; but here almost insuperable obstacles were before us. Independently of the idea of profanation of the body, the Egyptians have a theological doctrine that the dead feel the tortures to which they may be subjected.

"The pacha and the minister of war refused to undertake the responsibility of sanctioning the practice. At length, however, the chief of the Ulemas, a learned and enlightened man, though a devotee, was induced to connive at it. About two pupils were persuaded to commence on detached parts of the body. They were soon cured of their prejudices, and convinced of the indispensability of dissection: in turn, they convinced their parents and relatives; these instructed the rest of the people: at length, from mere toleration, we were afforded effectual encouragement, and Ibrahim Pacha and his ministers of state assisted at a lecture on practical anatomy.

[A general murmur of approbation here rose through the Academy. M. Clot then continued.]

"Our labours of translation were meanwhile persevered in, and we succeeded in accomplishing the version into Arabic of M. Magendie on Physiology, and M. Begin on Elementary Surgery. With regard to the nomenclature, I had recourse to the following expedient. A number of learned men were engaged and employed in collecting amidst oral traditions and written books, all the medical terms they could find. Five or six thousand words were thus obtained, and when it became necessary to invent a new one, these persons, in full academy, made choice of the new term. Five years thus passed away, when at length the murderous cholera burst over Egypt. The foreign physicians fled; in twenty-nine days Cairo, in a population of 260,000 persons, lost 60,000. Left alone in Cairo, I despatched all the pupils from the school. One of them now present was attached to the household of the pacha, and treated sixty patients with success. Of these 150 pupils, thirty perished by the epidemic, at the termination of which they returned to the school, from whence one hundred were soon called to accompany the expedition into Syria.

"To conclude: the necessity of native professors now became obvious. I proposed to the pacha, whose inexhaustible benevolence made all our undertakings less difficult, that a certain number of the most distinguished pupils should be sent to Europe, at the public expense, to study in the western schools, and bring back into their own country the knowledge acquired abroad. This proposal was at once agreed to. As for myself, the pacha requested that I should preserve in France the costume of the East. My rank has cost me no sacrifice of opinion or change of creed. The toleration of Mehemet is as boundless as his benevolence. As well as the rank of Bey, he has conferred on me that of Colonel, with the pecuniary appointments to the amount of 36,000 francs per annum."

Repeated acclamations testified the interest created in the French Academy by M. Clot's communication.—*Lancet.*

---

### MONTHLY LIST OF MEDICAL BOOKS.

### METEOROLOGICAL JOURNAL,

By Messrs. Harris and Co. Mathematical Instrument Makers, 50, High Holborn.

| | Moon. | Rain | Thermometer. | | | Barometer. | | De Luc's Hygrometer | | Winds. | | Atmospheric Variation. | | |
|---|---|---|---|---|---|---|---|---|---|---|---|---|---|---|
| Oct. | | gauge. | 9a.m. | max. | min. | 9a.m. | 10p.m. | 9a.m. | 10p.m. | 9 a.m. | 10p.m. | 9 a.m. | 2 p.m. | 10 p.m. |
| 20 | | | 45 | 54 | 46 | 30.10 | 30.12 | 77 | 77 | NNE | NNE | foggy | fine | fine |
| 21 | | | 51 | 57 | 44 | .08 | .03 | 79 | 79 | NE | E | fine | fine | cloudy |
| 22 | | | 47 | 53 | 49 | .02 | .07 | 80 | 78 | ESE | ENE | | | |
| 23 | ● | | 52 | 55 | 45 | .10 | .10 | 78 | 77 | ESE | ENE | foggy | | foggy |
| 24 | | | 50 | 53 | 47 | .12 | .15 | 77 | 78 | ESE | SE | | | cloudy |
| 25 | | | 50 | 53 | 47 | .20 | .18 | 78 | 80 | SE | SE | | | cloudy |
| 26 | | | 49 | 51 | 44 | .15 | .15 | 79 | 79 | SE | SE | | | foggy |
| 27 | | | 45 | 50 | 45 | .13 | .13 | 80 | 80 | SE | SE | | foggy | |
| 28 | | | 48 | 53 | 48 | .00 | 29.83 | 82 | 84 | SE | SW | | cloudy | cloudy |
| 29 | | | 53 | 57 | 45 | 29.76 | .78 | 84 | 82 | S | N | cloudy | foggy | fine |
| 30 | | | 50 | 59 | 49 | .95 | .85 | 82 | 83 | W | SW | foggy | fine | fine |
| 31 | ☽ | | 54 | 57 | 47 | .90 | .77 | 78 | 81 | WSW | SW | fine | | rain |
| *Nov.* | | | | | | | | | | | | | | |
| 1 | | | 52 | 58 | 50 | .54 | .64 | 79 | 81 | W | NNW | cloudy | | fine |
| 2 | | | 55 | 59 | 53 | .54 | .30 | 82 | 80 | SSW | WNW | | cloudy | cloudy |
| 3 | | .56 | 55 | 57 | 42 | .53 | .61 | 78 | 74 | NW | W | fine | fine | fine |
| 4 | | | 48 | 54 | 38 | .57 | .52 | 75 | 75 | W | SW | | | |
| 5 | | | 42 | 46 | 40 | .52 | .74 | 73 | 70 | N | N *va.* | | | |
| 6 | | .05 | 45 | 48 | 40 | 30.04 | 30.14 | 72 | 76 | NNE | NNE | | | |
| 7 | | | 44 | 47 | 40 | .18 | .12 | 76 | 75 | NE | NE | | | |
| 8 | ○ | | 41 | 44 | 38 | 29.84 | 29.72 | 77 | 78 | NE | NNE | rain | cloudy | cloudy |
| 9 | | .06 | 40 | 47 | 38 | .67 | .67 | 78 | 75 | SE | S | foggy | foggy | foggy |
| 10 | | | 45 | 49 | 43 | .49 | .22 | 79 | 80 | S | S | rain | rain | rain |
| 11 | | | 46 | 51 | 39 | .33 | .40 | 81 | 80 | SW | WSW | foggy | fine | cloudy |
| 12 | | .49 | 45 | 45 | 40 | .50 | .56 | 78 | 78 | WSW | W | | foggy | foggy |
| 13 | | | 41 | 48 | 41 | .59 | .57 | 76 | 74 | ENE | SE | | | |
| 14 | | | 47 | 51 | 49 | .52 | .56 | 77 | 78 | SSW | SW | fine | cloudy | cloudy |
| 15 | ☾ | | 50 | 50 | 39 | .69 | .86 | 78 | 76 | SSE | NNE | foggy | cloudy | cloudy |
| 16 | | | 41 | 50 | 41 | 30.07 | 30.16 | 75 | 77 | NE | NE | fine | fine | fine |
| 17 | | | 44 | 49 | 38 | .16 | .10 | 73 | 74 | NE | ESE | | | fine |
| 18 | | | 41 | 46 | 42 | 29.84 | 29.76 | 74 | 76 | | | foggy | foggy | cloudy |
| 19 | | | 46 | 48 | 45 | .71 | .64 | 79 | 80 | SSE | SSE | foggy | fine | cloudy |

The quantity of Rain fallen in October, was 72.100ths of an inch.

